# Development of an alkaliptosis-related lncRNA risk model and immunotherapy target analysis in lung adenocarcinoma

**DOI:** 10.3389/fgene.2025.1573480

**Published:** 2025-04-08

**Authors:** Xiang Xiong, Wen Liu, Chuan Yao

**Affiliations:** Department of Cardiothoracic Surgery, The Affiliated Hospital of Jiujiang University, Jiujiang, Jiangxi, China

**Keywords:** lung adenocarcinoma, alkaliptosis, lncRNAs, prognosis, immunotherapy sensitivity

## Abstract

**Background:**

Lung cancer has the highest mortality rate among all cancers worldwide. Alkaliptosis is characterized by a pH-dependent form of regulated cell death. In this study, we constructed a model related to alkaliptosis-associated long non-coding RNAs (lncRNAs) and developed a prognosis-related framework, followed by the identification of potential therapeutic drugs.

**Methods:**

The TCGA database was utilized to obtain RNA-seq-based transcriptome profiling data, clinical information, and mutation data. We conducted multivariate Cox regression analysis to identify alkaliptosis-related lncRNAs. Subsequently, we employed the training group to construct the prognostic model and utilized the testing group to validate the model’s accuracy. Calibration curves were generated to illustrate the discrepancies between predicted and observed outcomes. Principal Component Analysis (PCA) was performed to investigate the distribution of LUAD patients across high- and low-risk groups. Additionally, Gene Ontology (GO) and Gene Set Enrichment Analysis (GSEA) were conducted. Immune cell infiltration and Tumor Mutational Burden (TMB) analyses were carried out using the CIBERSORT and maftools algorithms. Finally, the “oncoPredict” package was employed to predict immunotherapy sensitivity and to further forecast potential anti-tumor immune drugs. qPCR was used for experimental verification.

**Results:**

We identified 155 alkaliptosis-related lncRNAs and determined that 5 of these lncRNAs serve as independent prognostic factors. The progression-free survival (PFS) and overall survival (OS) rates of the low-risk group were significantly higher than those of the high-risk group. The risk signature functions as a prognostic factor that is independent of other variables. Different stages (I–II and III–IV) effectively predict the survival rates of lung adenocarcinoma (LUAD) patients, and these lncRNAs can reliably forecast these signatures. GSEA revealed that processes related to chromosome segregation and immune response activation were significantly enriched in both the high- and low-risk groups. The high-risk group exhibited a lower fraction of plasma cells and a higher proportion of activated CD4 memory T cells. Additionally, the OS of the low TMB group was significantly lower compared to the high TMB group. Furthermore, drug sensitivity was significantly greater in the high-risk group than in the low-risk group. These lncRNAs may serve as biomarkers for treating LUAD patients.

**Conclusion:**

In summary, the construction of an alkaliptosis-related lncRNA prognostic model and drug sensitivity analysis in LUAD patients provides new insights into the clinical diagnosis and treatment of advanced LUAD patients.

## Introduction

Lung cancer has the highest mortality rate worldwide, with lung adenocarcinoma (LUAD) accounting for the majority of cases. Most patients are diagnosed at an advanced stage, missing optimal treatment opportunities and facing a very low survival rate (Ren et al., 2024; Siegel et al., 2024). Numerous treatment methods are available for LUAD. In addition to surgical resection, recent advancements have seen the widespread use of radiotherapy, chemotherapy, immunotherapy, and targeted therapy (Miao et al., 2023; Barr et al., 2024; Makarem and Jänne, 2024; Liu et al., 2024; Xu and Lu, 2025). Despite the rapid development of these treatment modalities, their efficacy for advanced LUAD remains limited. However, by predicting risk scores, we can identify potential therapeutic targets. Consequently, a more comprehensive treatment model could lead to improved treatment outcomes. In summary, the establishment of a prognostic model may provide clinicians with innovative insights.

Alkaliptosis a pH-dependent form of regulated cell death and represents a novel strategy for cancer treatment across various tumor types (Liu et al., 2019). This process can be activated through the upregulation of nuclear factor-kappa B (NF-κB) pathways, which is followed by the downregulation of carbonic anhydrase 9 (CA9) (Liu et al., 2019; Park et al., 2023). This may benefit from ACSS2-mediated acetyl-coenzyme A production and subsequent histone acetylation (Que et al., 2023). Numerous studies have established a connection between alkaliptosis and various conditions, including ovarian cancer, diabetic cardiomyopathy (DCM), gastric cancer, and diabetic kidney disease (DKD) (Liu et al., 2019; Park et al., 2023; Que et al., 2023; Yu et al., 2024). The latest researches indicate that alkaliptosis-related genes play an important role in LUAD (Yu et al., 2024; Wei et al., 2023). This underscores the significance of developing treatments that specifically target the molecular mechanisms associated with alkaliptosis.

Long non-coding RNA (lncRNA) refers to a category of non-coding RNA transcripts that exceed 200 nucleotides in length (Hao et al., 2023; Srinivas et al., 2024). These molecules are involved in regulating the gene expression of various malignant tumors in human tissues, but the specific mechanisms are not yet clear. A large number of studies have shown that LncRNA can regulate autophagy in Acute kidney injury (AKI) through multiple regulatory pathways, thereby affecting the pathological development of AKI (Wang et al., 2023). This kind of autophagy mediated by regulating the expression of specific signaling pathways and related genes also plays an important role in bone destruction in rheumatoid arthritis (RA) (Lei et al., 2023). In addition, lncRNA was found to be involved in the regulation of iron metabolism and affected the development of breast cancer by hyperactivating YAP (He et al., 2023). Abnormal 5-methylcytosine (m5C) methylation lncRNA NR_033928 also plays an important role in the development and prognosis of gastric cancer by interacting with the IGF2BP3/HUR complex (Fang et al., 2023). Notably, specific lncRNA LINC01614 promotes LUAD progression by affecting glutamate metabolism in cancer cells (Liu et al., 2022). In addition, lncRNA FAM83A-AS1 was found to affect glucose metabolism in LUAD cells both *in vivo* and *in vitro* (Chen Z. et al., 2022). However, we still do not know enough about the specific role of lncRNA in LUAD.

In summary, the study of alkaliptosis-related lncRNA holds significant importance for the diagnosis and treatment of LUAD. In this research, we first established the framework for alkaliptosis-related lncRNA, developed a prognosis-related model, and validated the reliability of this model. Finally, we conducted further screening for potential therapeutic drugs targeting LUAD. This work offers a novel perspective on the management of LUAD.

## Materials and methods

### Data acquisition

The Cancer Genome Atlas (TCGA) database (https://portal.gdc.cancer.gov/) was utilized to collect RNA-sequencing (RNA-seq) data, clinical information, and mutation data from 59 normal individuals and 541 patients with LUAD. Samples with ambiguous staging or survival data were excluded. Raw counts were normalized using DESeq2, as described in Smith et al. (Conesa et al., 2016). To identify potential alkaliptosis-related lncRNAs, co-expression correlation analysis was conducted using the limma software package with criteria set at |R| > 0.4 and P < 0.001 on the expression of lncRNAs and alkaliptosis-related genes.

### Identification of alkaliptosis-related lncRNAs

Recent studies on alkaliptosis have identified key genes (Conesa et al., 2016; Qiu et al., 2024; Wang et al., 2022). We randomly divided 541 LUAD samples into two groups at a one-to-one ratio (Qiu et al., 2024). In the training group, we utilized multivariate Cox regression analysis to identify alkaliptosis-related lncRNAs. By conducting Lasso-Cox regression on significantly expressed lncRNAs, we pinpointed 13 alkaliptosis-related lncRNAs. Penalty parameter (λ) was selected via 10-fold cross-validation to minimize partial likelihood deviance (Wang et al., 2022). The most promising lncRNA (P < 0.05) was identified through multivariable Cox regression analysis. The optimal model parameters were utilized to create features, followed by the calculation of the risk score using the formula: Risk score = β lncRNA1 × Exp lncRNA1 + β lncRNA2 × Exp lncRNA2 + β lncRNA3 × Exp lncRNA3 + ••• + β lncRNAn × Exp lncRNAn.

### Independent prognostic analysis and survival analysis

The prognostic model was developed using the training group and validated using the testing group. This model provided a formula to calculate the risk score for each sample. Based on the median risk score, samples were categorized into high- and low-risk groups. Utilizing the “survival” package, overall survival (OS) and progression-free survival (PFS) of LUAD patients were analyzed in these high- and low-risk groups. Univariate and multivariate independent prognostic analyses were conducted to assess the independent prognostic value of clinical features. Risk scores were utilized to generate a visualization of LUAD patients’ survival time and lncRNAs expression using the “pheatmap” package. Additionally, the 1-, 3-, and 5-year area under the ROC curve (AUC) of clinical features in the training, testing, and combined groups were calculated with the “survival ROC” package.

### Construction of nomogram and calibration curves

Nomograms were generated using the “survival” and “rms” packages, incorporating variables such as age, gender, T stage, TNM stage, N stage, and risk score. Calibration curves were utilized to visualize the disparities between predicted and actual outcomes based on the nomogram. Subsequently, survival analysis was conducted on patients with LUAD at stages I–II and stages III–IV.

### Analysis of principal component and functional enrichment

The distribution of high-risk and low-risk groups of LUAD patients was analyzed using the “limma” and “scatterplot3d” software packages for PCA. Additionally, the clusterProfiler software package was utilized to conduct Gene Ontology (GO) and Gene Set Enrichment Analysis (GSEA) on alkaliptosis-related lncRNA, resulting in obtaining p values for both the high-risk and low-risk groups. Significant increases were observed in the risk group, with an incidence rate <0.05 and a false discovery rate (FDR) <0.05.

### Analysis of immune cell infiltration and TMB

The CIBERSORT algorithm was initially employed to estimate the abundance of 22 immune cells in each sample, which were calculated using the LM22 signature matrix, following the protocol by Newman et al. (Newman et al., 2015). Subsequently, values with a p-value <0.05 were filtered for the final analysis. Visualization of the 22 immune cell types was conducted using the “parblot” package in R language, while control analysis between low- and high-risk groups was performed using the “vioplot” package. The “maftools” package was utilized to assess the relationship between TMB and high and low risk. Furthermore, survival analysis was carried out using appropriate software to determine the association between high- and low-TMB groups and survival rates in LUAD patients, with statistical significance set at P < 0.05.

### Immunotherapy and immune drug analysis

The relationship curve between TMB and risk score (p < 0.05) was obtained using the “ggpubr” and “limma” packages. Additionally, anti-tumor immune drugs and their treatment sensitivity were predicted using “oncoPredict,” “ggplot2,” and “ggpubr” packages with pFilter = 0.001 and corPvalue = 0.001. The Genomics of Drug Sensitivity in Cancer (GDSC) database was obtained from oncoPredict’s Open Science Framework at https://osf.io/c6tfx/.

### Cell culture and treatment

LUAD cell lines (A549, H1299) and normal bronchial epithelial cells (BEAS-2B) were cultured in RPMI-1640 medium supplemented with 10% fetal bovine serum (FBS) and 1% penicillin/streptomycin at 37°C with 5% CO_2_. These cells were purchased from Fuheng Biotechnology (Shanghai, China). Then, cells were treated with anti-PD-1 antibody (10 μg/mL, Pembrolizumab) or 5-fluorouracil (50 μM) for 48 h (Herbst et al., 2016; Zitvogel et al., 2010).

### RNA extraction and qPCR

Total RNA was extracted using TRIzol reagent (Invitrogen) and reverse-transcribed into cDNA using the PrimeScript RT Reagent Kit (Takara). qPCR was performed using SYBR Green Master Mix (Roche) on a QuantStudio 6 Flex system. Primers for the five lncRNAs (LINC00707, AC092718.4, MHENCR, AP005137.2, AC092143.3) and the reference gene GAPDH were designed using Primer-BLAST (sequences in Supplementary Table S1). Cycling conditions: 95°C for 10 min, followed by 40 cycles of 95°C for 15 s and 60°C for 1 min. Relative expression was calculated using the 2^(−ΔΔCt) method.

### Statistical analysis

Statistical analyses were performed using R software (version 4.1.3) in the research. Data are presented as mean ± SD from three independent experiments. Differences between groups were analyzed using one-way ANOVA followed by Tukey’s post-hoc test. P < 0.05 was considered significant.

## Results

### Identification of alkaliptosis -related lncRNAs

**FIGURE 1:**
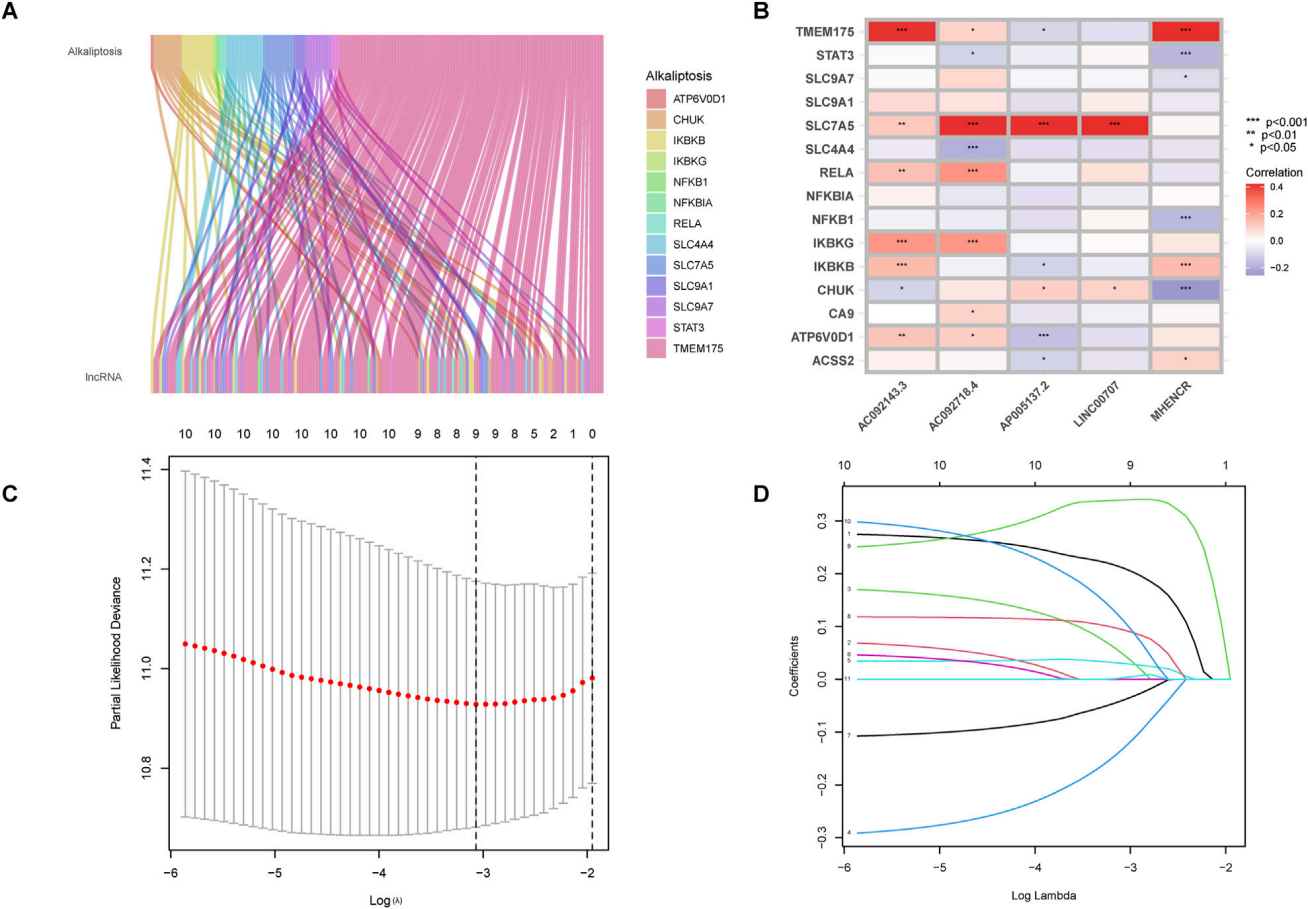
Identification of alkaliptosis-related lncRNAs. **(A)** The Sankey diagram showed the co-expression relationship of alkaliptosis-related genes and alkaliptosis-related lncRNAs. **(B)** Heatmap showed the relationship between alkaliptosis-related genes and alkaliptosis-related lncRNAs. **(C)** Cross-validation of LASSO regression. **(D)** Trajectory of each independent variable. *, *p* < 0.05; **, *p* < 0.01; ***, *p* < 0.001.

The RNA-seq data of LUAD patients from TCGA was used to differentiate between mRNAs and lncRNAs by referencing gene annotation files. Among 16,877 lncRNAs, 155 alkaliptosis-related lncRNAs were identified along with 13 alkaliptosis-related genes (|R| >0.4 and P < 0.001). A Sankey diagram (Figure 1A) was utilized to visualize the co-expression relationships between the alkaliptosis-related genes and lncRNAs. Co-expressed lncRNAs (AC092718.4, AP005137.2, and AP005137.2) strongly correlate with SLC7A5 (P < 0.001), suggesting their role in pH regulation. MHENCR shows positive correlation with TMEM175 (P < 0.001), indicating potential resistance to alkaliptosis. The study utilized Lasso Cox regression analysis to identify alkaliptosis-related lncRNAs. Univariate and multivariate Cox analysis revealed 11 lncRNAs and 5 lncRNAs as independent prognostic factors, respectively. Subsequently, a risk score was calculated using these 5 lncRNAs (refer to Figures 1C, D). The risk score formula was determined as follows: Risk score = (0.289519080381684 * LINC00707) + (0.229206032056953 * AC092718.4) + (−0.329442435225535 * MHENCR) + (0.41395459437268 * AP005137.2) + (0.328943252027341 * AC092143.3). Additionally, the correlation heatmap (Figure 1B) visually depicted the relationship between alkaliptosis-related genes and lncRNAs.

### Survival analysis

**FIGURE 2:**
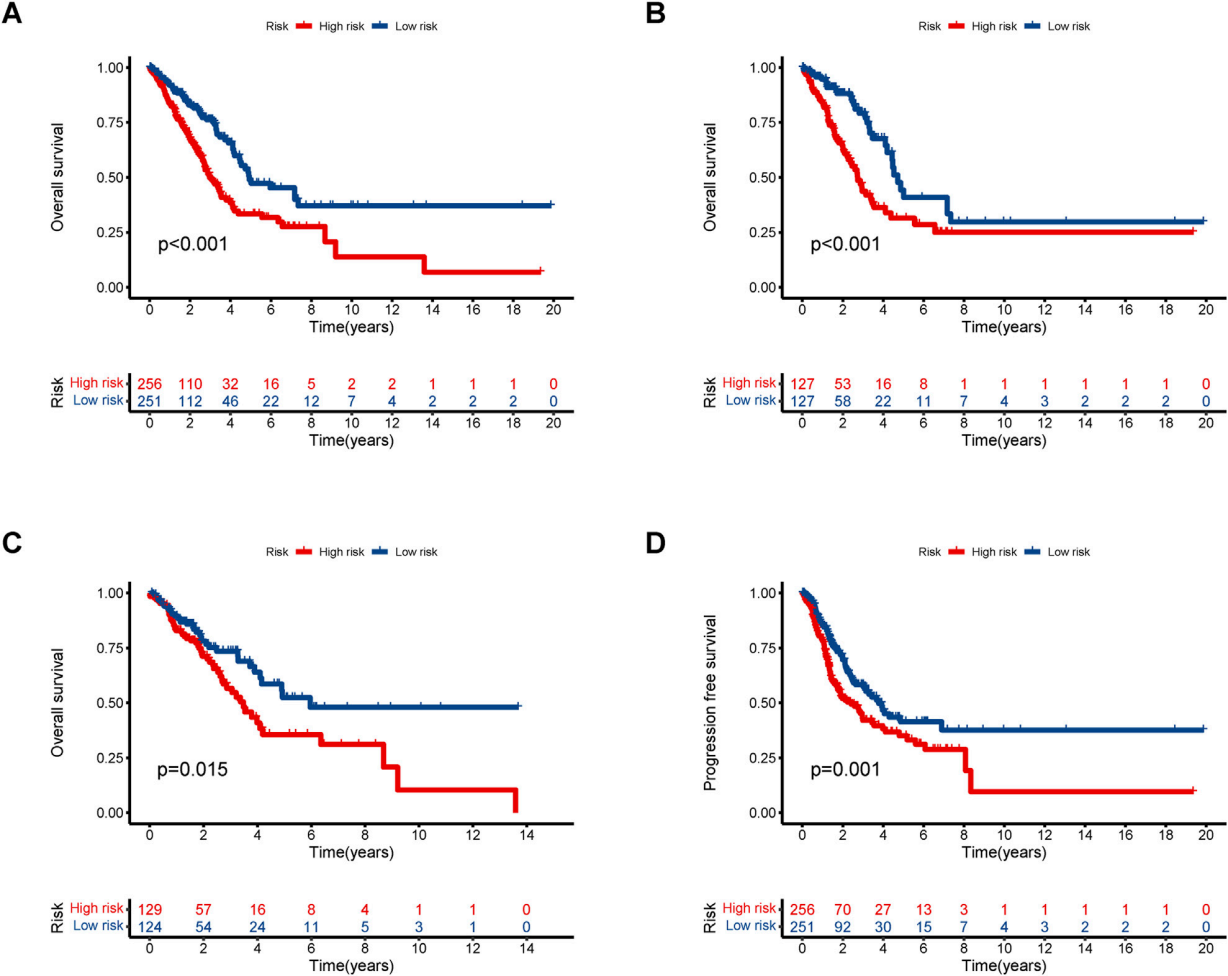
Kaplan–Meier survival analyses of LUAD patients. We divided patients into high-risk and low-risk groups based on median risk and predicted overall survival (OS) and progression-free survival (PFS) for each group. **(A)** OS in all groups. **(B)** OS in the training groups. **(C)** OS in the testing groups. **(D)** PFS in all groups.

**FIGURE 3:**
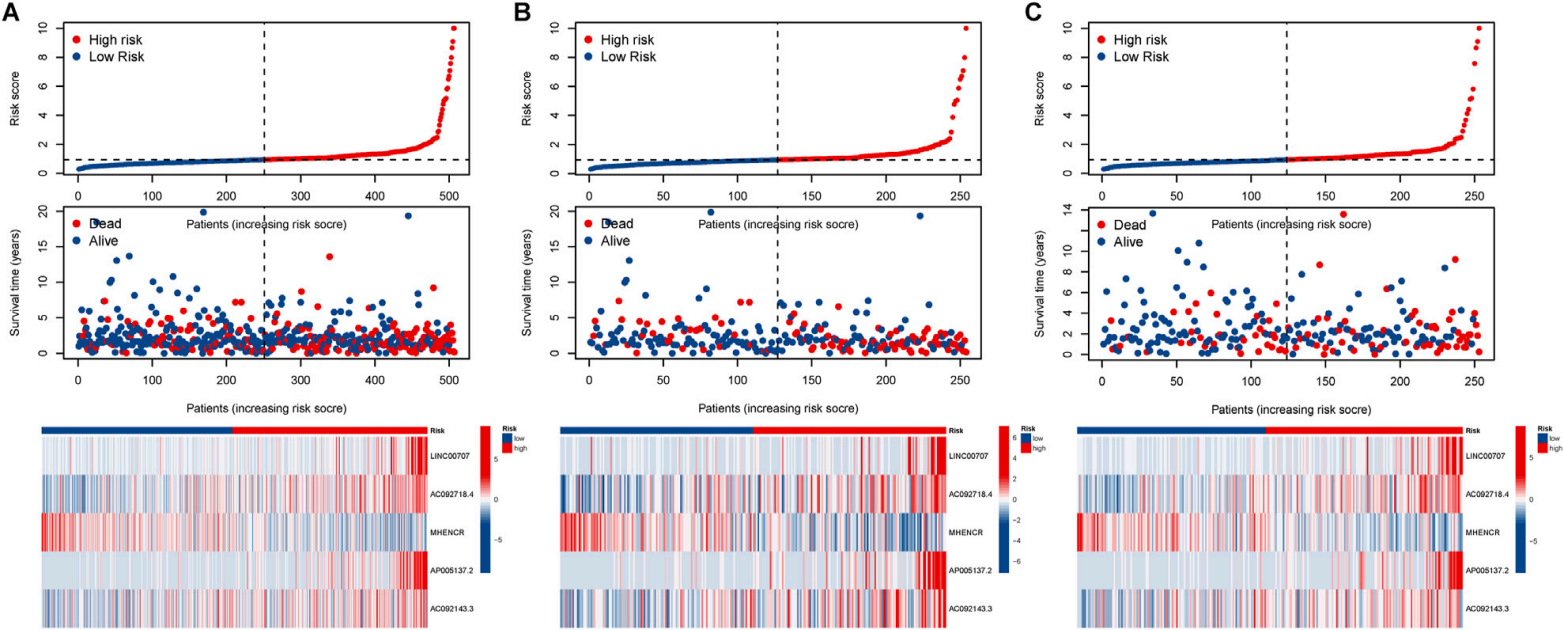
Assessment of the prognostic value of alkaliptosis-related lncRNA models. Survival curves of high-risk and low-risk LUAD patients; Heatmap showed high-and low-risk levels of 5 lncRNAs in the **(A)** all group, **(B)** training group, and **(C)** testing groups.

Based on the median risk, patients were categorized into high and low-risk groups. In all groups, including training and testing, the progression-free survival (PFS) and overall survival (OS) of the low-risk group were significantly higher than those of the high-risk group (Figures 2A–D). Analysis of the risk curve revealed that the mortality rate of patients in the low-risk group was notably lower compared to the high-risk group. Additionally, a heatmap displayed the high and low-risk levels of 5 lncRNAs, with MHENCR representing low-risk lncRNAs and LINC00707, AC092718.4, AP005137.2, and AC092143.3 representing high-risk lncRNAs (Figures 3A–C).

### Independent prognostic factors analysis

**FIGURE 4:**
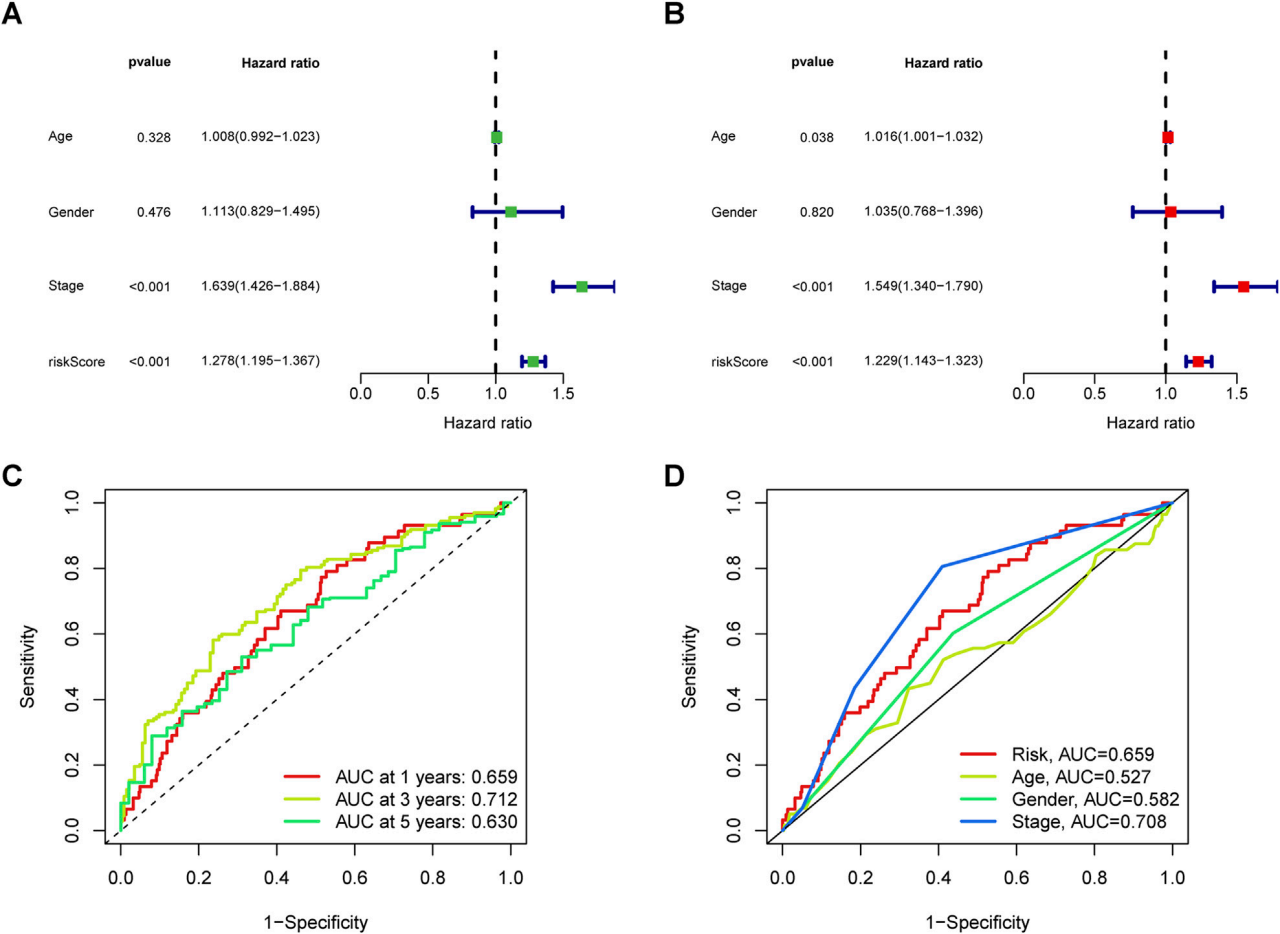
Analysis of the independent prognostic value of clinical features. **(A)** Univariate and **(B)** multivariate independent prognostic analysis to determine whether clinical characteristics are independently associated with OS. **(C)** 1-, 3-, and 5-year area under the ROC curve (AUC) of clinical indicators in all groups. **(D)** AUC values of clinical indicators.

Univariate Cox regression results indicated a significant association between stage (hazard ratio [HR] = 1.639, 95% CI 1.426–1.884; P < 0.001) and risk score (HR = 1.278, 95% CI 1.195–1.367; P < 0.001) (Figure 4A). Moreover, Multivariate Cox regression analysis revealed that both stage (HR = 1.549, 95% CI 1.340–1.790; P < 0.001) and risk score (HR = 1.229, 95% CI 1.143–1.323; P < 0.05) independently influenced overall survival, underscoring the prognostic value of the risk signature irrespective of other variables (Figure 4B). ROC curves results indicated that the stage (AUC = 0.708) outperformed other factors such as age (AUC = 0.527), gender (AUC = 0.582), and risk score (AUC = 0.659) (Figure 4D), supporting its utility in stratifying high-risk patients for aggressive therapies. Combining risk scores with TNM staging improved predictive accuracy. Across all groups, the AUC values for 1-, 3-, and 5-year overall survival rates were 0.659, 0.712, and 0.630, respectively (Figure 4C), demonstrating the reliability of our model.

### Clinical characteristics analysis and PCA

**FIGURE 5:**
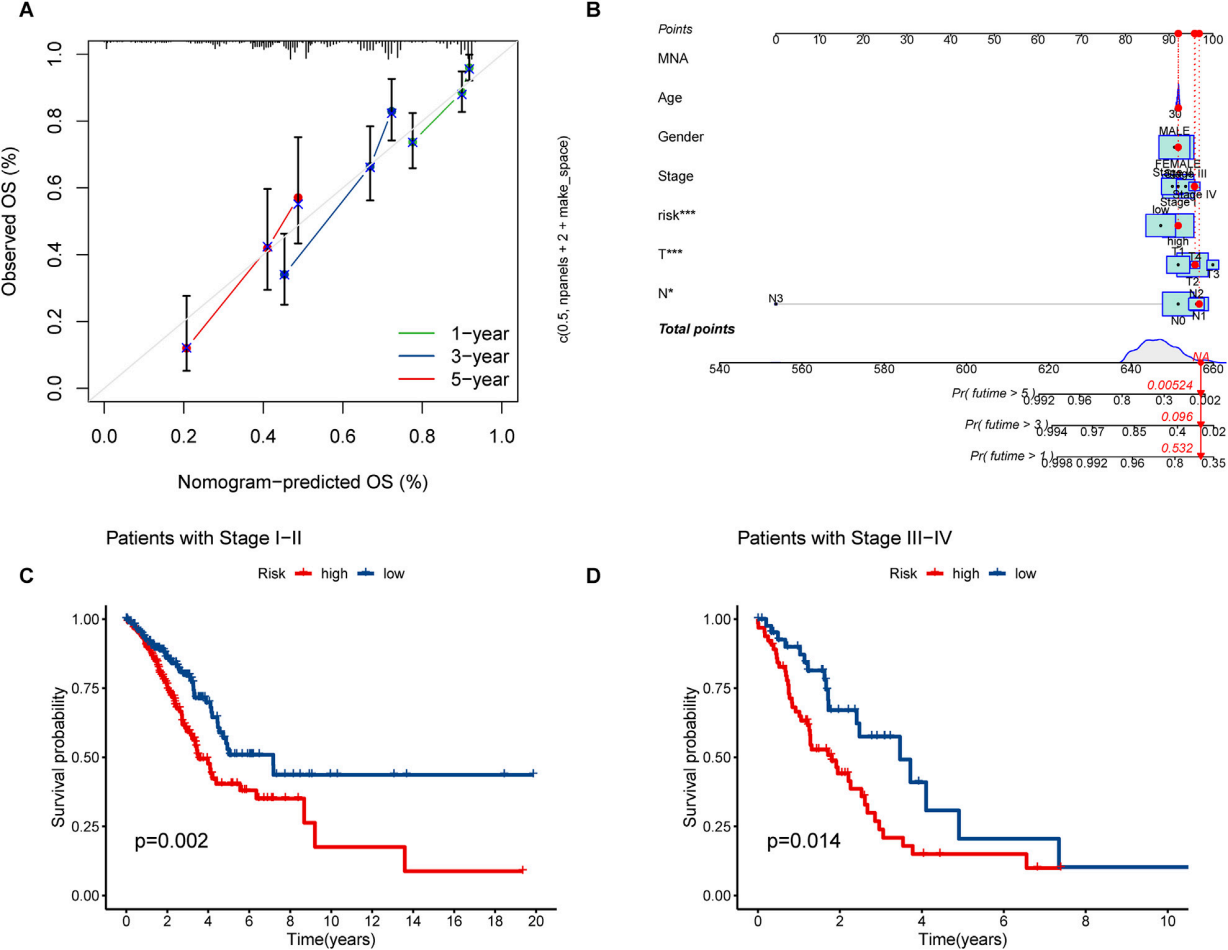
Nomograms and survival curves were used to predict OS and survival in LUAD patients. **(A)** Calibration curves for 1-year, 3-year, and 5-year. **(B)** Prognostic nomogram for OS of LUAD patients. **(C, D)** Survival analyses of LUAD Patients at stages I–II and stages III–IV.

**FIGURE 6:**
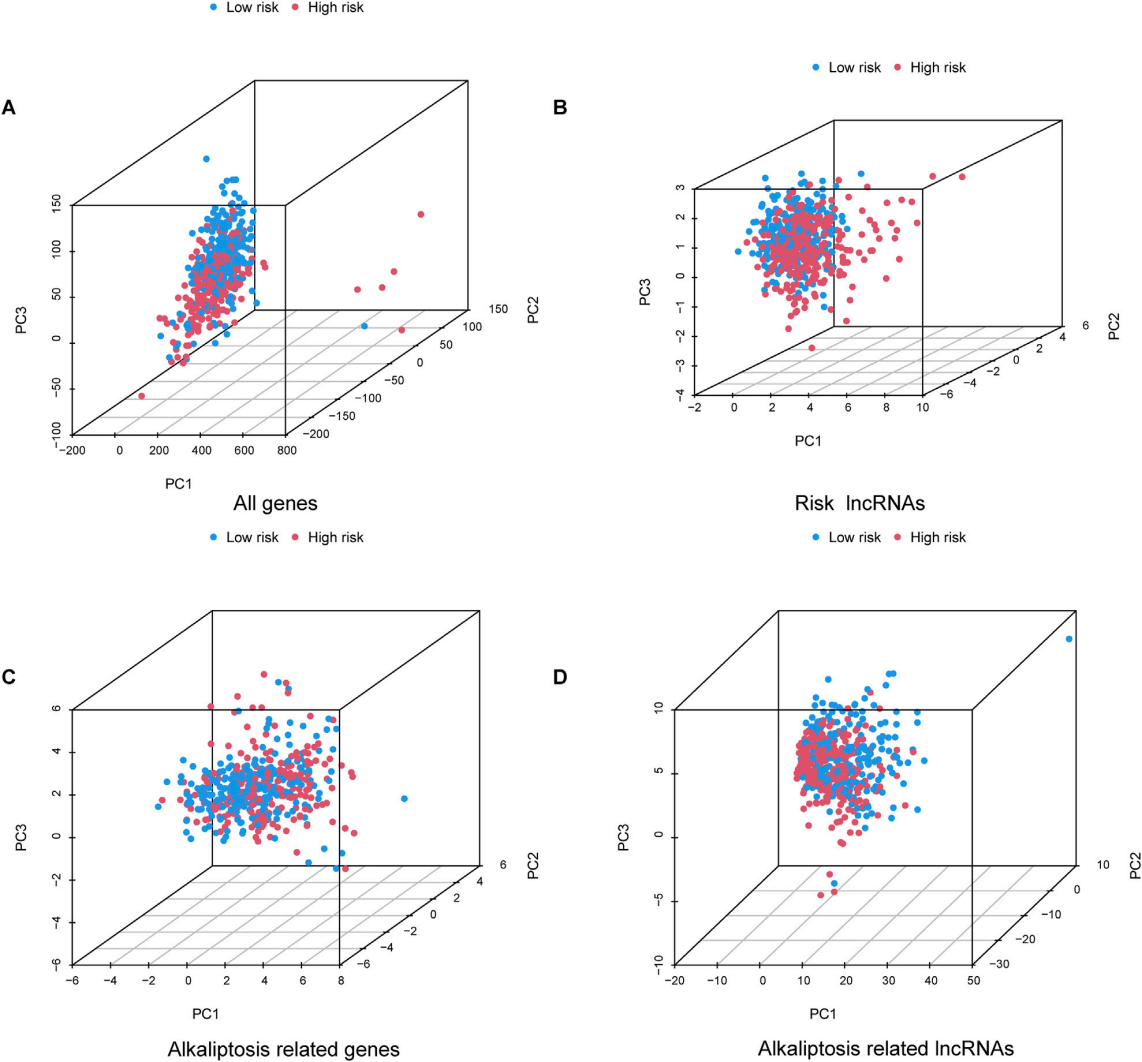
Principal component analysis. PCA observed the distribution of **(A)** All genes, **(B)** Risk lncRNAs, **(C)** Alkaliptosis-related genes, and **(D)** Alkaliptosis-related lncRNAs.

The nomogram we developed accurately predicted the 1-year, 3-year, and 5-year overall survival (OS) of LUAD patients based on factors such as age, gender, T stage, TNM stage, N stage, and risk score (Figures 5A, B). Furthermore, our analysis of high and low-risk groups at different stages (I-II and III-IV) demonstrated the predictive ability of stages in determining survival rates for LUAD patients (Figures 5C, D). Principal component analysis (PCA) revealed the distribution of all genes, risk lncRNAs, alkaliptosis-related genes, and alkaliptosis-related lncRNAs, indicating their potential in predicting these signatures reliably (Figures 6A–D).

### Analysis of functional enrichment and immune-related function

**FIGURE 7:**
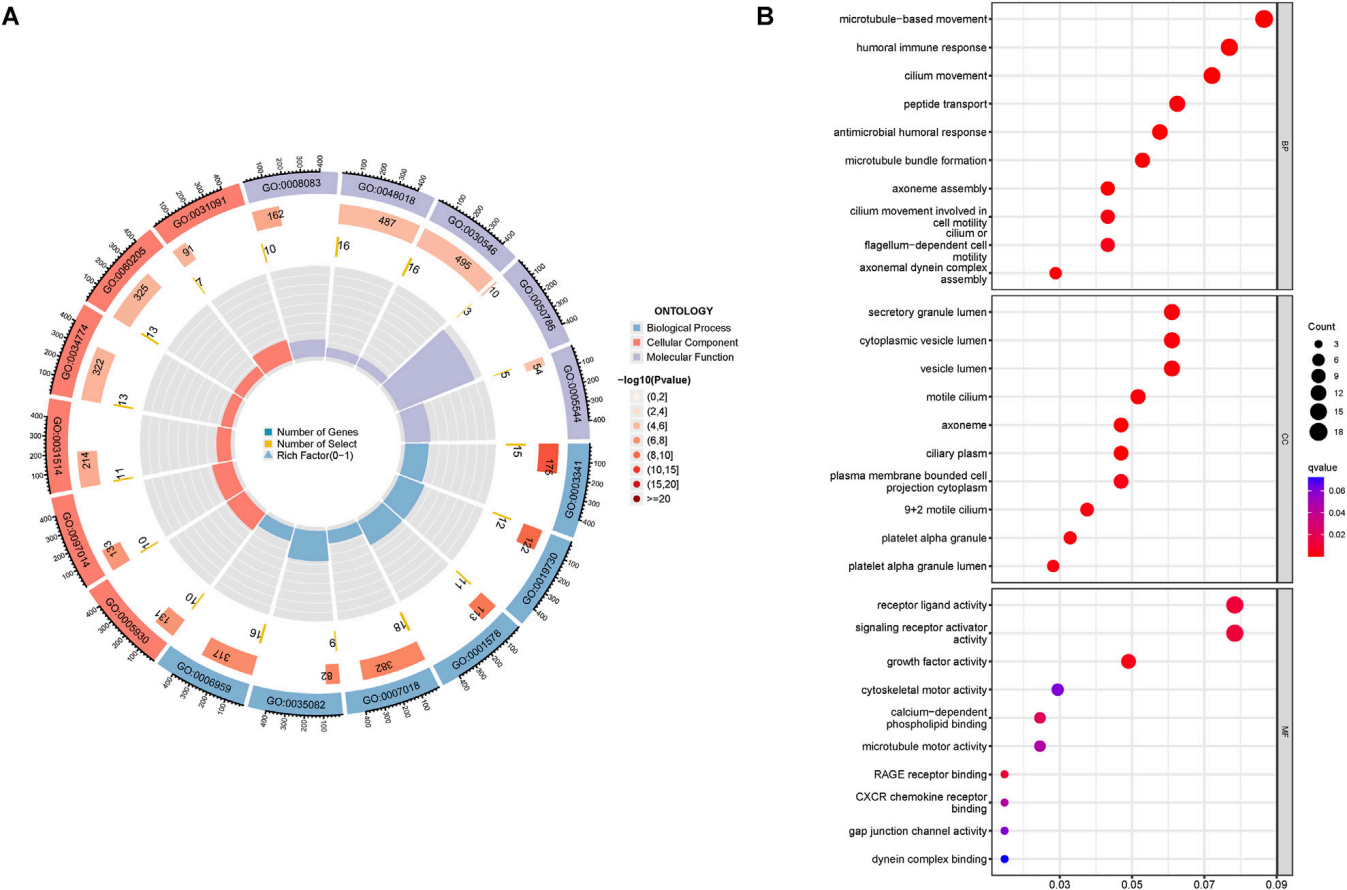
Functional enrichment analysis. GO enrichment analyses of the alkaliptosis-related lncRNAs were shown by **(A)** circle diagram and **(B)** bubble chart.

**FIGURE 8:**
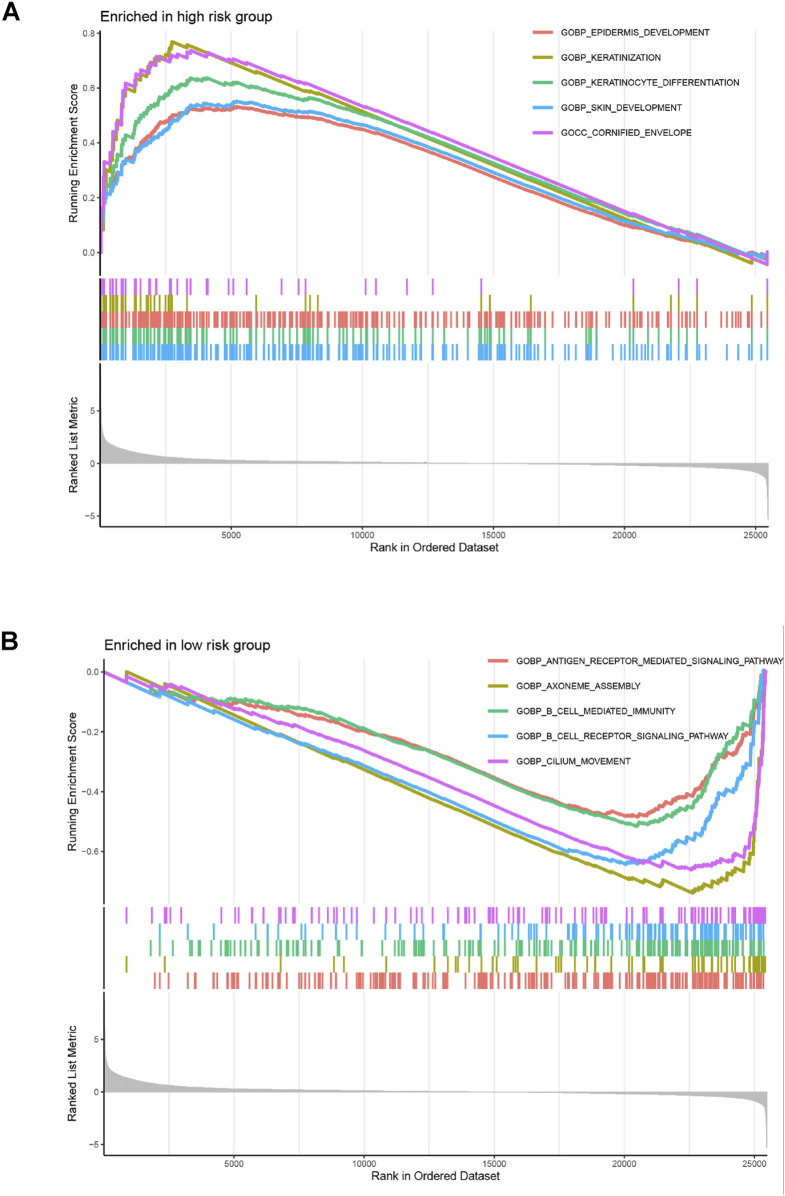
GSEA enrichment analyses. GSEA enrichment analyses of the alkaliptosis-related lncRNAs in **(A)** high-risk group and **(B)** low-risk group.

**FIGURE 9:**
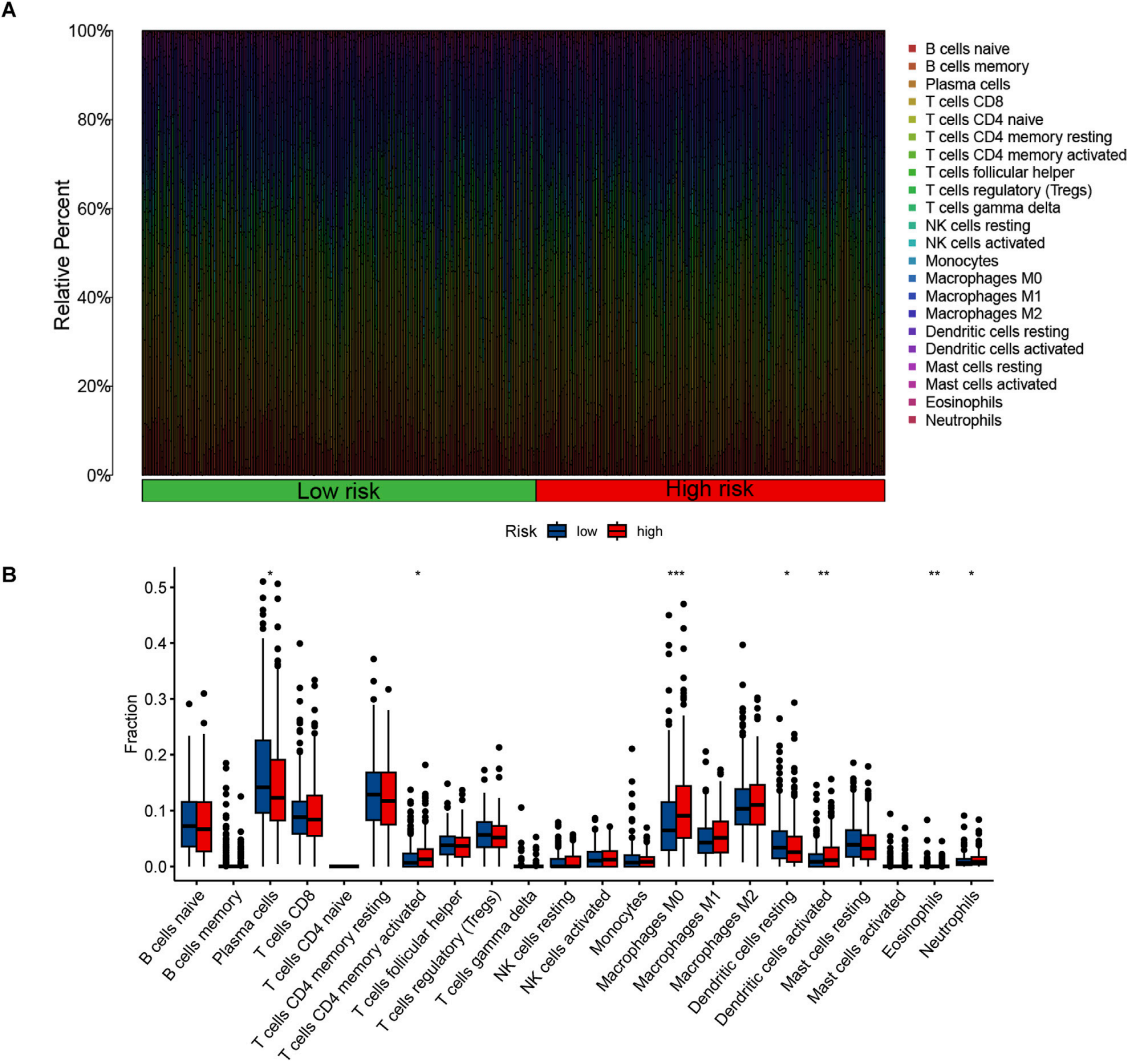
The proportions and differences of 22 types of immune cells in high-risk and low-risk groups were analyzed. **(A)** The percentage histogram illustrates the relative percentages of immune cells. **(B)** The violin plot displays the differences in immune cell infiltration fractions between high-risk and low-risk LUAD patients, with low-risk samples represented in blue and high-risk samples in red. *, *p* < 0.05; **, *p* < 0.01; ***, *p* < 0.001.

The GO analysis results indicated that alkaliptosis-related lncRNAs were enriched in microtubule-based movement, humoral immune response, and cilium movement (Figures 7A, B). GSEA analysis results revealed that epidermis development, keratinization, keratinocyte differentiation, skin development, and cornified envelope were the top five significantly enriched processes in the high-risk group. Conversely, in the low-risk group, antigen receptor-mediated signaling pathway, axoneme assembly, B cell-mediated immunity, B cell receptor signaling pathway, and cilium movement were the top five significantly enriched processes (Figures 8A, B). The study analyzed the distribution of 22 different types of immune cells in high-risk and low-risk groups using percentage histograms. It was found that the high-risk group had a higher percentage of macrophages compared to the low-risk group (Figure 9A). Additionally, the violin plot analysis revealed that the high-risk group had a lower fraction of plasma cells and resting dendritic cells compared to the low-risk group (p < 0.05). On the other hand, the high-risk group exhibited a higher proportion of activated T cells CD4 memory, macrophages M0, activated dendritic cells, and neutrophils compared to the low-risk group (p < 0.05 for all) (Figure 9B).

### TMB analysis

**FIGURE 10:**
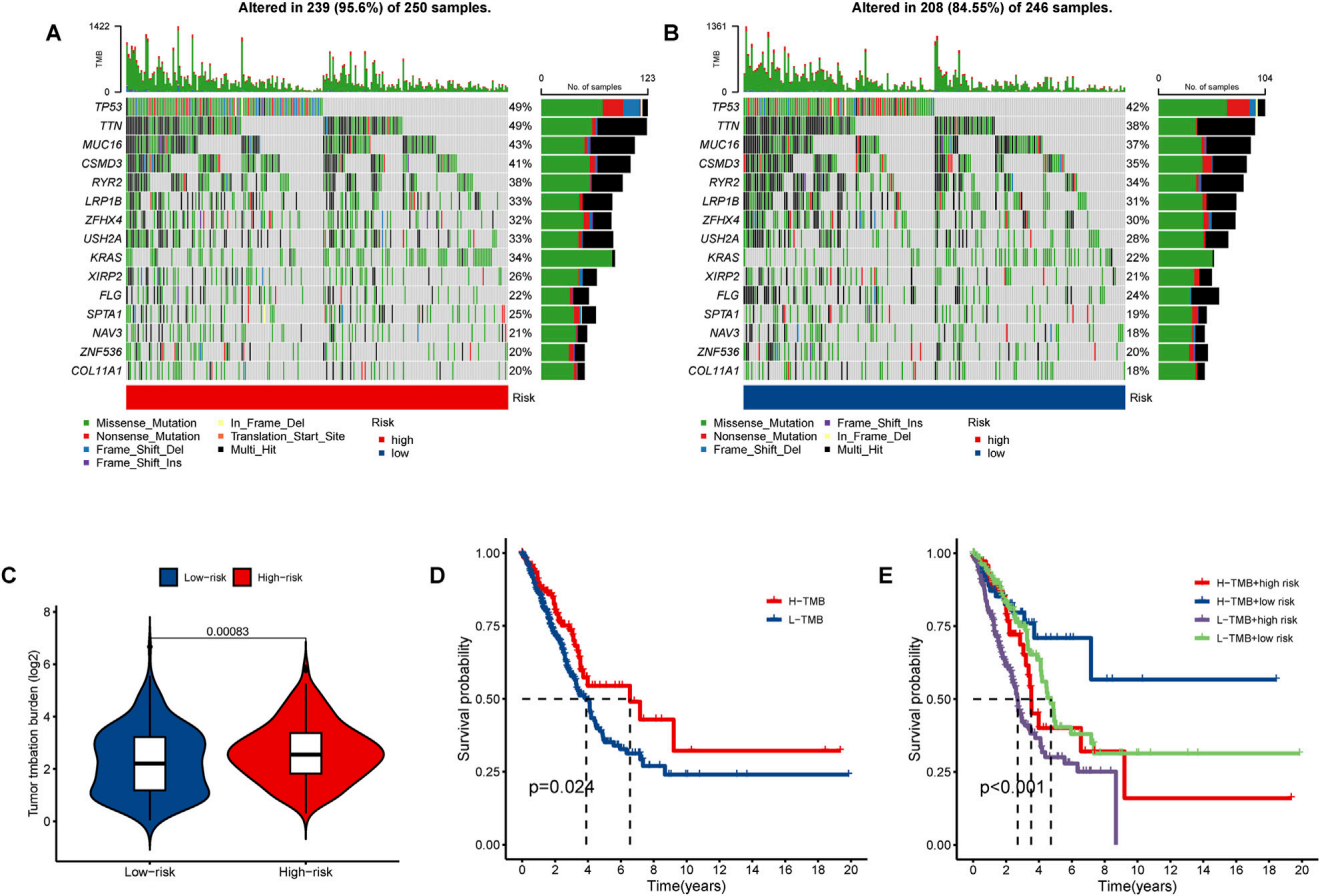
The tumor mutational burden (TMB) and somatic mutation frequencies were analyzed in high- and low-risk lung adenocarcinoma (LUAD) patients. The waterfall plot illustrates the top fifteen mutated genes in LUAD patients for the **(A)** high-risk group (250 samples) and **(B)** low-risk group (246 samples). **(C)** The violin plot presents the TMB results. **(D)** Kaplan-Meier curves demonstrate the differences in overall survival (OS) between LUAD patients with high and low TMB. **(E)** Survival curves for LUAD patients are shown based on varying TMB and risk scores.

Mutation analysis revealed a notable discrepancy in mutation frequency between the low-risk and high-risk groups as determined by the maftools algorithm. Specifically, TP53 mutations were observed in 42% of the low-risk group compared to 49% in the high-risk group, TTN mutations were found in 38% of the low-risk group and 49% in the high-risk group, and MUC16 mutations were present in 37% of the low-risk group and 43% in the high-risk group (Figures 10A, B). Furthermore, a significant variation in tumor mutational burden (TMB) was detected between the low-risk and high-risk cohorts (Figure 10C). Notably, the overall survival (OS) of patients with low TMB was markedly lower than those with high TMB, as illustrated in Figure 10D. Patients in the high TMB and low-risk category exhibited the most favorable prognosis, whereas those in the low TMB and high-risk category had the poorest prognosis (Figure 10E).

### Drug sensitivity analysis

**FIGURE 11:**
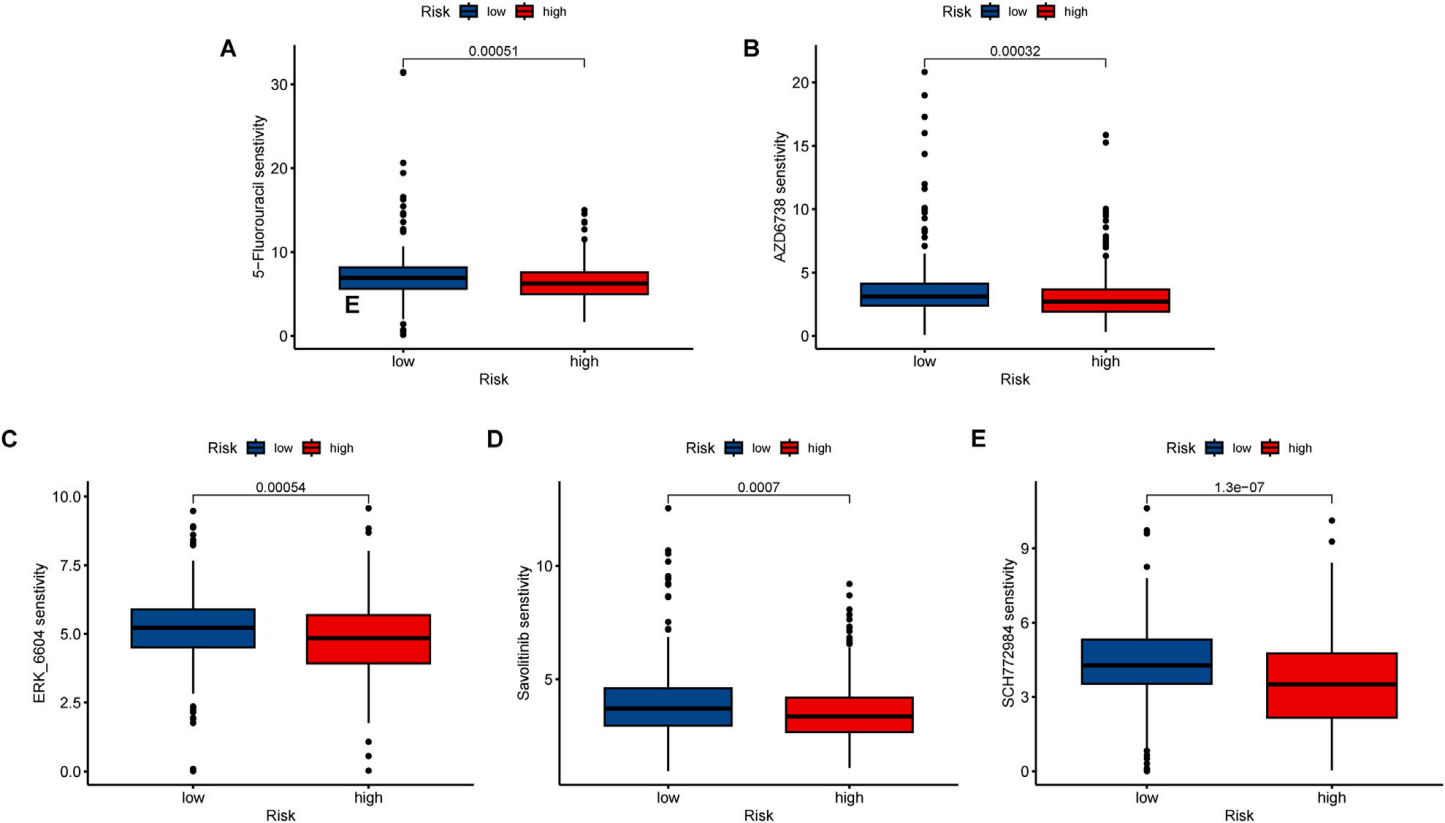
Immunotherapy and drug sensitivity analysis. Different drug sensitivity of **(A)** 5-fluorouracil, **(B)** AZD6738, **(C)** ERK_6604, **(D)** Savolitinib, and **(E)** SCH772984. ***, *p* < 0.001.

The “oncoPredict” package was utilized to identify potentially effective anti-tumor drugs for LUAD patients, such as 5-fluorouracil, AZD6738, ERK_6604, Savolitinib, and SCH772984. Analysis of the IC50 values (concentration that inhibits cell growth by 50%) in Figures 11A–E revealed that the high-risk group exhibited significantly greater drug sensitivity compared to the low-risk group.

### Immunotherapy effects on lncRNAs expression

**FIGURE 12:**
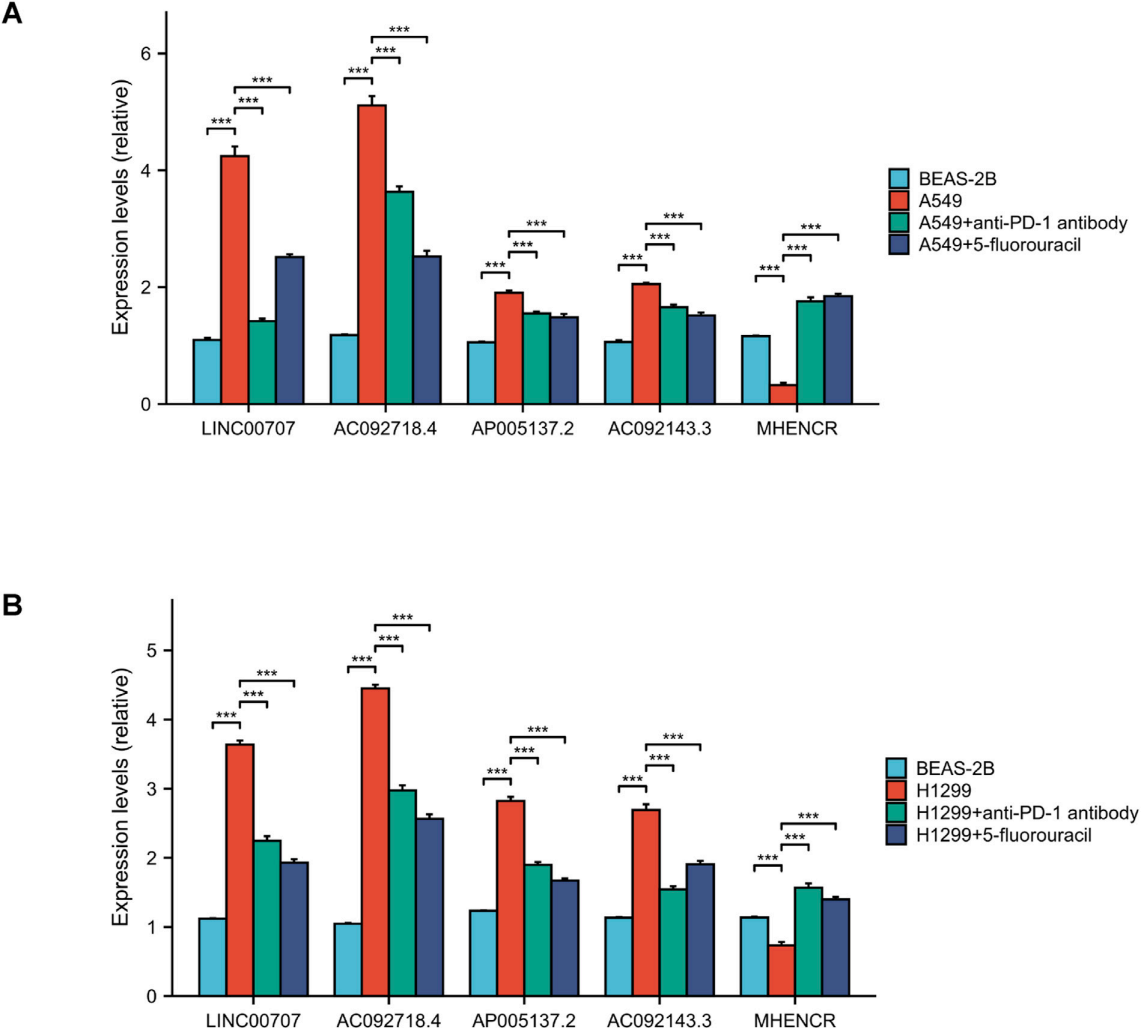
lncRNA expression and drug verification. **(A, B)** qPCR analysis of 5 lncRNAs in LUAD vs normal cells. And the outcomes of anti-PD-1 antibody and 5-fluorouracil on lncRNA expression. ***, *p* < 0.001.

The expression levels of high-risk lncRNAs, specifically LINC00707, AC092718.4, AP005137.2, and AC092143.3, were significantly elevated in A549 and H1299 cells compared to BEAS-2B (p < 0.001). Conversely, the expression levels of the low-risk lncRNA MHENCR in LUAD cells were found to be lower than those in BEAS-2B (p < 0.001). Following treatment with anti-PD-1 antibodies, the expression levels of high-risk lncRNAs exhibited a significant decrease (p < 0.001), while low-risk lncRNAs demonstrated a 2- to 6-fold increase (p < 0.001). Similar patterns were observed after treatment with 5-Fluorouracil, where high-risk lncRNAs were downregulated and low-risk lncRNAs were upregulated (p < 0.001) (Figures 12A, B).

## Discussion

Lung adenocarcinoma remains a prevalent form of lung cancer worldwide, particularly in its late stages, which are associated with a poorer prognosis and lower survival rates (Newman et al., 2015). Current lung cancer treatment strategies emphasize early detection, including screening with low-dose computed tomography, although this approach has its limitations. Therefore, identifying key prognostic factors is crucial for improving early diagnosis of lung adenocarcinoma (Herbst et al., 2016). Long non-coding RNA (lncRNA) is a functional genetic element consisting of over 200 nucleotides that does not encode proteins but plays a role in various cellular processes (Bridges et al., 2021). lncRNA CERS6-AS1 promotes lung adenocarcinoma progression and drug resistance by upregulating ANLN expression through sponge miR-424-5p (Ting et al., 2024). Alkaliptosis, a pH-dependent process of cell death initiated by the small molecule compound JTC801, has emerged as a novel therapeutic strategy for treating malignancies. The two primary signaling pathways involved in this process are the NF-κB-CA9 pathway and the ATP6V0D1-STAT3 pathway, both of which contribute to cellular alkaliptosis (Chen et al., 2023a; Chen et al., 2023b; Song et al., 2018; Zhu et al., 2021). Recently, Fang et al. found that the HMGB1-AGER-STING1 pathway exerts biological effects during alkaliptosis by inhibiting cytokine production (Fang et al., 2021). Recent studies indicate that macropinocytosis, an endocytic process, promotes resistance to alkalization in human pancreatic ductal adenocarcinoma (PDAC) cells by uptake of extracellular substances (Chen et al., 2024). Studies have shown that Arenobufagin (ArBu) and Cisplatin (CDDP) jointly inhibit the growth of tumor cells *in vivo* and *in vitro* by inducing alkaliptosis (Liu et al., 2023). However, the mechanism of alkaliptosis-related lncRNAs in LUAD needs further exploration.

In our study, Lasso Cox regression analysis identified lncRNAs associated with alkaliptosis. A total of five prognostic alkaliptosis-related lncRNAs—MHENCR, LINC00707, AC092718.4, AP005137.2, and AC092143.3—were identified through multivariate Cox regression analysis, leading to the construction of a prognostic model. ROC analysis, survival rate comparisons, nomogram evaluations, and pheatmap visualizations indicated that the prognostic characteristics of these five alkaliptosis-related lncRNAs effectively differentiated between high-risk and low-risk groups, as well as between stages I–II and III–IV patients, thereby accurately predicting outcomes for LUAD patients. These lncRNAs serve as independent prognostic factors, distinct from other common clinical features. Among the five alkaliptosis-related lncRNAs identified in the LUAD context, MHENCR, LINC00707, AC092718.4, AP005137.2, and AC092143.3 were found to be involved in cancer. Chen et al. confirmed that MHENCR is a new therapeutic target for melanoma, which is achieved by activating the PI3K-Akt pathway mediated by miR-425/489 (Chen et al., 2017). In addition, new research results confirm that the LINC00707 lncRNA-mediated miR-382-5p/VEGFA pathway plays an important role in cervical cancer (Guo et al., 2021). LINC00707 can also promote the development of hepatocellular carcinoma (HCC) by activating the ERK/JNK/AKT signaling pathway (Wang et al., 2019). Furthermore, LINC00707 promotes Triple-negative breast cancer (TNBC) disease progression through the March2/mTOR pathway mediated by competitive binding to miR-423-5p (Li et al., 2024). Studies have found that LINC00707 reduces rheumatic heart disease (RHD) myocardial fibrosis and regulates immune dysfunction through the miR-145-5p/S1PR1 pathway (Zhao et al., 2023). LINC00707 potentially enhances NF-κB signaling, promoting CA9 downregulation and intracellular alkalinization, thereby triggering alkaliptosis. Conversely, MHENCR may suppress the STAT3 pathway, which is known to counteract alkaliptosis resistance. These lncRNAs likely interact with miRNAs or proteins to regulate key pathways, such as ACSS2-mediated acetyl-CoA production, which influences histone acetylation and alkaliptosis sensitivity. It has been confirmed that knocking down the expression of lncRNA AC092718.4 can inhibit the growth of LUAD cells and accelerate cell apoptosis (Chen S. et al., 2022). Furthermore, the GO and GSEA pathway analysis indicated that alkaliptosis-related lncRNAs may be related to LUAD development.

We subsequently analyzed the relationship between TMB and risk scores in patients with LUAD. Our findings indicated that patients categorized in the high TMB and low-risk group exhibited the most favorable prognosis. TMB has been established as an effective biomarker for predicting the efficacy of immunotherapy across various cancer types. Recent studies have shown that TMB is considered a reliable biomarker by determining the total number of mutations in each coding region of the tumor genome (Gabbia and De Martin, 2023; Marabelle et al., 2020; Palmeri et al., 2022; Wang X. et al., 2024). TMB in mouse lung cancer models with deleted or mutated p53 genes promotes immune resistance, such as reduced antigen presentation (Zhu et al., 2023). In addition, different tumors also have different tumor burdens. For example, breast tumors and gliomas have lower TMB (Alsaihati et al., 2021). Our results indicate that patients with a high TMB exhibit a lower survival rate (P < 0.05). Compared to existing lncRNA models (Lu et al., 2021), our model demonstrates superior specificity for alkaliptosis and stronger correlation with TMB. Additionally, the expression levels of TP53, TTN, and MUC16 are elevated in high-risk patients. Mutations in the tumor suppressor gene TP53 contribute to tumorigenesis, making the treatment of mutated TP53 a critical therapeutic strategy (Hassin and Oren, 2022). TP53, a tumor suppressor gene located on chromosome 17p13.1, plays a complex biological role in the cell cycle (Pereira et al., 2023). The mutation frequency of TP53 is different in different types of cancer, and the mutation frequency of solid tumors is higher than that of MNs including acute myeloid neoplasm (AML), myeloproliferative neoplasm (MPN), and myelodysplastic syndrome (MDS) (Zhao et al., 2024). Titin (TTN) is a sarcomeric protein that forms the myofibrillar skeleton and regulates muscle disorders and cardiomyopathy (Jolfayi et al., 2024). Recent studies demonstrate that truncating TTN mutations promote the development of congenital myopathies in centronuclear myopathies (CNM) (Ceyhan-Birsoy et al., 2013). In addition, Han et al. found that TTN gene mutations play a potential role in thyroid cancer (THCA) and may indicate a poor prognosis (Han et al., 2022).

In this study, we found that the high-risk group show significantly greater drug sensitivity than the low-risk group. However, the sensitivity of high- and low-risk LUAD patients to immunotherapy requires further analysis. Immune checkpoint inhibitors (ICIs), tumor vaccines, chimeric antigen receptors T cells, oncolytic viruses, and bispecific antibodies showed great potential in tumor immunotherapy (Li et al., 2015; Ye et al., 2024; Li et al., 2023; Shen et al., 2024). A HARMONi-5 study demonstrated the safety and efficacy of the bispecific antibody ivonescimab as first- or second-line monotherapy in patients with advanced or metastatic non-small cell lung cancer (NSCLC) (Wang L. et al., 2024). The latest research findings indicate that administering the Janus kinase 1 (JAK1) inhibitor itacitinib following anti-PD-1 (programmed cell death protein 1) immunotherapy enhances immune function and the anti-tumor response in mice (Mathew et al., 2024). Notably, pembrolizumab in combination with chemoradiation significantly improved progression-free survival (PFS) in patients with newly diagnosed locally advanced cervical cancer (Lorusso et al., 2024). As cutting-edge technologies such as scRNAseq, spatial transcriptomics, and high-parameter flow cytometry become more widely used in the future, they provide a basic guarantee for our understanding of the role of immune cells in the tumor microenvironment (TME) (Donisi et al., 2024). We utilized the “oncoPredict” package to identify potentially effective anti-tumor drugs for patients with LUAD, which have been extensively employed in cancer-related treatments. Our findings indicate that drug sensitivity in the high-risk group is significantly greater than that in the low-risk group. We clarified the clinical relevance of the identified drugs in LUAD. AZD6738 (ATR inhibitor) synergizes with PARP inhibitors in TP53-mutant LUAD, while SCH772984 (ERK inhibitor) targets MAPK pathway activation common in KRAS-mutant LUAD. However, these predictions require validation in preclinical models, such as patient-derived xenografts, to assess efficacy and toxicity. Limitations include the lack of pharmacokinetic data and tumor microenvironment heterogeneity in silico models (Capasso et al., 2024). LINC00707, AC092718.4, AP005137.2, and AC092143.3 are oncogenic lncRNAs in LUAD, as their high baseline expression correlates with tumor aggressiveness. Immune checkpoint inhibitors (anti-PD-1) and 5-Fluorouracil modulate lncRNA expression, suggesting their role in LUAD drug response. MHENCR acts as a tumor suppressor, upregulated by treatment, potentially enhancing alkaliptosis sensitivity. However, the underlying mechanisms of action of these drugs in LUAD patients warrant further investigation.

## Conclusion

In this study, we developed a LUAD-specific alkaliptosis-related lncRNA model and validated its accuracy using risk scores. Furthermore, we conducted analyses on TMB and the sensitivity to immunotherapy, providing new insights for survival prediction and treatment strategies for clinical LUAD patients.

## Limitations

Our research still primarily relies on retrospective TCGA data, lacking a prospective cohort. Additionally, the mechanistic link between lncRNAs and alkalinization requires experimental validation. We need to further utilize LUAD organoids for functional studies to test the effects of lncRNA knockout. And evaluate the clinical trial of AZD6738 in high-risk LUAD patients.

## Data Availability

The datasets presented in this study can be found in online repositories. The names of the repository/repositories and accession number(s) can be found in the article/Supplementary Material.
