# Supplementary material for: Development of an alkaliptosis-related lncRNA risk model and immunotherapy target analysis in lung adenocarcinoma
Source: Front Genet. 2025 Apr 8;16:1573480. doi: 10.3389/fgene.2025.1573480 (PMC12011837; doi:10.3389/fgene.2025.1573480)
Supplement: Supplementary file 1 [file Table1.docx]

**Supplementary Table S1** | Sequences of primers used in the study.

| lncRNA | Forward(5’-3’) | Reverse(5’-3’) |
| --- | --- | --- |
| LINC00707 | **CAGGTGCTGGTGAAGTTGCT** | **TGGTCACATGGTCCAGGTAG** |
| AC092718.4 | **GCTACCTGGAGCCATCAACT** | **CCTCACAGGGTCTCCATTGT** |
| MHENCR | **AGGACCTGGTGGAGATGCTG** | **TCCAGGTGCTCATCAGTTCC** |
| AP005137.2 | **TGCCTGGATTCTGGTCAATG** | **CTGTCACAGGGTCCTGTGTC** |
| AC092143.3 | **AGCTGGACCTCAACCTGAAC** | **GGTCCAGGTAGTGGCTGTCT** |

| Gene | Forward(5’-3’) | Reverse(5’-3’) |
| --- | --- | --- |
| GAPDH | **GGAGCGAGATCCCTCCAAAAT** | **GGCTGTTGTCATACTTCTCATGG** |
